# Trends in cross-border and illicit tobacco purchases among people who smoke in England, 2019–2022

**DOI:** 10.1136/tc-2023-057991

**Published:** 2023-07-18

**Authors:** Sarah E Jackson, Sharon Cox, Jamie Brown

**Affiliations:** 1 Behavioural Science and Health, UCL, London, UK

**Keywords:** Socioeconomic status, Surveillance and monitoring, Illegal tobacco products, Cessation

## Abstract

**Objectives:**

The last 5 years have seen substantial changes in England’s social and economic landscape as a result of Brexit, the COVID-19 pandemic and cost of living crisis. We aimed to examine changes in cross-border and illicit tobacco purchasing over this period.

**Design:**

Nationally representative monthly cross-sectional survey.

**Setting:**

England, 2019–2022.

**Participants:**

11 232 adults (≥18 years) who smoked in the past year.

**Main outcome measures:**

We estimated time trends in the proportion reporting purchasing tobacco from (1) cross-border and (2) illicit sources in the past 6 months.

**Results:**

Between February 2019 and October 2022, there was a non-linear increase in the proportion of participants reporting cross-border tobacco purchases (from 5.2% to 16.1% overall; prevalence ratio (PR)=3.10, 95% CI 2.03–4.73). Prevalence first increased from 5.2% to 15.4% between February 2019 and April 2020, before falling to 7.8% between April 2020 and September 2021 during the COVID-19 pandemic, and then increasing again to 16.1% by the end of the period. Changes in cross-border tobacco purchasing were more pronounced among participants from more advantaged (from 6.6% to 23.3%; PR=3.52, 95% CI 2.05–5.91) compared with less advantaged (4.4% to 11.5%; PR=2.61, 95% CI 1.17–5.20) social grades (p_interaction_=0.034). There was no overall change in the proportion reporting illicit tobacco purchases (from 9.2% to 8.5%; PR=0.92, 95% CI 0.70–1.21), nor any significant difference in trends by social grade (p_interaction_=0.783).

**Conclusions:**

Despite a fall in cross-border tobacco purchasing during the first year of the COVID-19 pandemic among adults in England who smoke, the proportion reporting cross-border tobacco purchases is now three times higher than it was at the start of 2019. The proportion reporting illicit tobacco purchases has not changed substantially.

**WHAT IS ALREADY KNOWN ON THIS TOPIC**

- Tobacco tax avoidance and evasion strategies, such as buying tobacco cheaply from cross-border or illicit sources, undermine the effectiveness of tax policy.

**WHAT THIS STUDY ADDS**

- Whether, and if so to what extent, cross-border and illicit tobacco purchases among adults in England who smoke have changed in the context of recent events, including Brexit, the COVID-19 pandemic and the ongoing cost of living crisis.

**HOW THIS STUDY MIGHT AFFECT RESEARCH, PRACTICE OR POLICY**

- Among adults in England who smoke, cross-border tobacco purchases have tripled from February 2019 to October 2022, while the proportion reporting illicit tobacco purchases remains similar.

## Introduction

 Raising tobacco taxes is effective for reducing smoking prevalence and tobacco consumption^1 2^ and inequalities in smoking.^3 4^ Tobacco tax avoidance and tax evasion strategies undermine the effectiveness of tax policy by allowing access to cheaper tobacco. People who buy cigarettes from low/untaxed sources—and those who switch to cheaper tobacco—are less likely to try to quit smoking than those who continue to pay the full amount of tax.^5 6^ Understanding how use of these strategies is changing over time is important for informing policy.

Tax avoidance strategies include purchasing tobacco legally from low-tax jurisdictions across international borders, or duty free while travelling between countries (‘cross-border purchases’).^7^ Tax evasion strategies include obtaining tobacco from illegal sources where no tax is paid, such as smuggling or buying counterfeit (‘illicit purchases’).^8 9^ Between 2002 and 2014, 12%–20% of UK adults who smoke reported having last purchased cigarettes from a low or untaxed source.^10^ The majority (≥75% in most years) of this group (8%–16% of all those who smoke) reported cross-border purchases, with just 16%–33% (2.6%–3.7%) buying from illicit sources (eg, local sellers/friends/relatives).^10^ However, patterns differ by socioeconomic position: those from less advantaged groups are more likely to use discount/generic brands or hand-rolled tobacco, while those from more advantaged groups are more likely to purchase duty-free tobacco (presumably because they are more likely to travel overseas).^11^

Several factors may have affected the availability and use of illicit and cross-border tobacco in England in recent years. First, as a result of Brexit, people travelling from the UK to countries within the European Union (EU) have been able to bring back duty-free cigarettes. The UK officially left the EU on 31 January 2020, but entered a transition period for the rest of 2020 during which trade, travel and freedom of movement remained largely unchanged.^12^ Changes to duty-free shopping, including tobacco, were implemented in January 2021.^13^ Second, these changes in duty-free purchasing were accompanied by a concomitant reduction in the quantities of relatively cheap duty-paid tobacco products travellers are permitted to import from the EU for personal use (from up to 800 cigarettes, 400 cigarillos, 200 cigars or 1 kg of tobacco pre-Brexit to 200 cigarettes, 100 cigarillos, 50 cigars or 250 g of tobacco post-Brexit). Given tobacco prices in frequently visited countries like Spain and Greece are significantly lower than the UK,^14^ this reduction in the tobacco allowance may have had a substantial impact on cross-border purchasing habits (although full border controls with the EU have yet to be applied^15^ so the extent to which these limits are currently being enforced is not clear). Third, the COVID-19 pandemic (from March 2020) restricted social interaction and international travel, which may have reduced access to cheap tobacco. Finally, the pandemic and, more recently, the ongoing cost of living crisis (since late 2020) have exposed many people to financial hardship as a result of loss of earnings^16^ and the cost of everyday essentials and household bills rising faster than average incomes.^17 18^ This may have increased motivation to reduce the cost of tobacco among those who smoke,^19 20^ particularly among less advantaged socioeconomic groups (eg, those on a low income).^11 20^

This study aimed to examine changes in reported purchasing of (1) cross-border and (2) illicit tobacco between 2019 and 2022 among adults in England who smoke, and to compare differences by occupational social grade. This aim was addressed via a regression analysis of data from the Smoking Toolkit Study, a nationally representative survey of adults in England.

## Method

### Design

The Smoking Toolkit Study is a nationally representative monthly cross-sectional survey in England.^21 22^ It uses a hybrid of random probability and simple quota sampling to select a new sample of ~1700 adults (≥18 years) each month. Full details are provided elsewhere.^22^

Data were collected face to face up to February 2020. However, restrictions under the COVID-19 pandemic meant no data were collected in March 2020, and data since April 2020 were collected via telephone. The two data collection modalities use the same sampling and weighting approach and show good comparability.^23^,^25^

The present study used data from respondents who had smoked in the past year, analysing changes between February 2019 (a year before the UK left the EU) and October 2022 (the most recent data available on source of purchase at the time of analysis).

### Measures

Source of purchase was assessed with the question: ‘In the last 6 months, have you bought any cigarettes or hand-rolled tobacco from any of the following?’. Participants could select multiple responses from a list of options. *Cross-border purchasing* was coded 1 for those who reported buying cigarettes or tobacco abroad, or having friends/family buy abroad on their behalf, else it was coded 0. Duty-free sources within the UK were not specified as a response option and some respondents may have included these in their definition of cross-border sources. *Purchase from illicit sources* was coded 1 for those who reported buying cigarettes or tobacco under the counter (from newsagent/off-licence/corner shop), in a pub (somebody comes around selling cheap), from people who sell cheap cigarettes on the street, from people in the local area who are a trusted source of cheap cigarettes or cheap from friends, else it was coded 0. Source of purchase was assessed in all monthly waves up to April 2022, then reduced to quarterly assessment (July and October 2022) due to funding changes.

*Social grade* was categorised based on National Readership Survey classifications^26^ as ABC1, which includes managerial, professional and upper supervisory occupations; and C2DE, which includes manual routine, semiroutine, lower supervisory and long-term unemployed.

### Statistical analysis

Data were weighted to match the population in England for age, social grade, region, housing tenure, ethnicity and working status within sex.^21^ Analyses were conducted in R V.4.2.2. The analysis plan was pre-registered (https://osf.io/eapcw/). We made two amendments: data on source of purchase were only collected until October 2022 and the associations between source of purchase and quitting outcomes were cut following peer review but are reported online (https://osf.io/eapcw/).

We used logistic regression to estimate monthly time trends in the proportion of respondents purchasing (1) cross-border and (2) illicit tobacco in the past 6 months. For the overall analysis, models only included time (survey month) as a predictor. For the social grade-specific analysis, models included time, social grade and their interaction as predictors. Survey month was modelled using restricted cubic splines. We had planned to use three knots but visual inspection of the modelled estimates against raw quarterly data points indicated the model did not provide a good fit for trends in cross-border tobacco purchasing. We therefore reran the models for both cross-border and illicit tobacco purchasing using four knots and compared the model fit using the Akaike information criterion (AIC; see online supplemental material). The criteria for selecting the best fitting model were either the model with the lowest AIC or the simplest model if within two units of the model with the lowest AIC. Our interpretation was based on the best fitting model for each outcome: four knots for cross-border and three knots for illicit. Prevalence ratios for changes across the whole time series (October 2022 vs February 2019) are presented, alongside 95% CIs calculated using bootstrapping. Corresponding data for any cheap tobacco purchasing (ie, cross-border and illicit combined) are provided in the online supplemental material.

## Results

Between February 2019 and October 2022, a total of 71 993 adults aged ≥18 were surveyed in England, of whom 12 432 (17.3%) reported having smoked in the past year. We excluded 1200 surveyed in waves that did not assess source of tobacco purchasing (May/June/August/September 2022), leaving a final sample of 11 232 adults who had smoked in the past year (46.2% female; mean (SD) age 41.8 years (16.7); 58.4% social grade C2DE).

**Figure 1:**
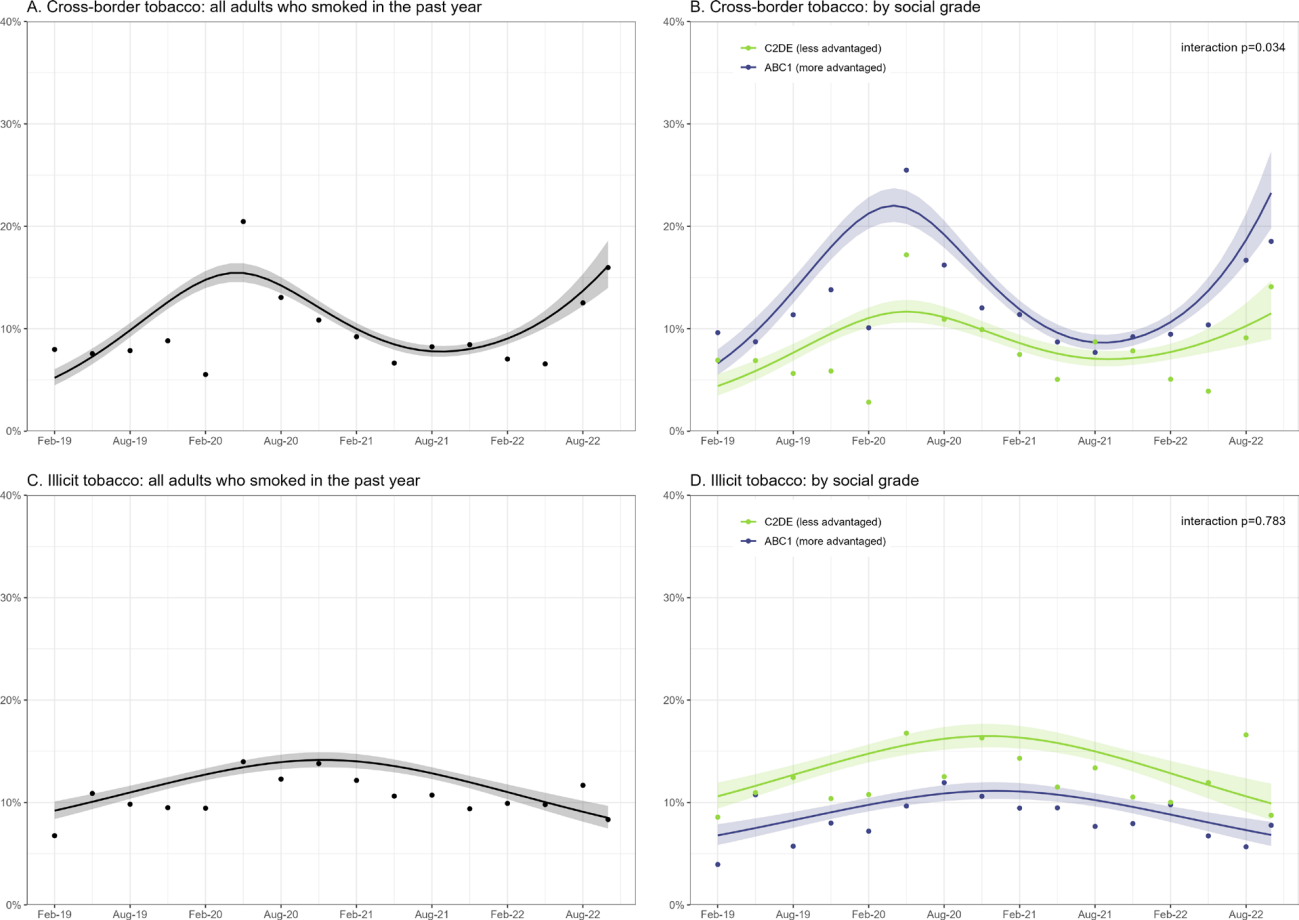
Percentage of adults in England who smoked in the past year and who reported purchasing cross-border and illicit tobacco, February 2019 to October 2022. Data are presented for all adults who smoked in the past year (A, C) and by social grade (B, D). Lines represent point estimates from logistic regression with survey month modelled non-linearly using restricted cubic splines (with four knots for cross-border tobacco and three knots for illicit tobacco; see online supplemental material for details of model selection). Shaded areas represent SEs. Points represent raw weighted prevalence by quarter (see online supplemental material for figures showing raw weighted prevalence by month).

From February 2019 to October 2022, the proportion of respondents reporting cross-border tobacco purchases increased from 5.2% to 16.1% (table 1). The increase over time was not linear: modelled estimates showed prevalence increased from 5.2% to 15.4% between February 2019 and April 2020 (raw weighted data points indicated prevalence was relatively stable until a sharp increase in Q2-2020); prevalence then fell to 7.8% between April 2020 and September 2021, before increasing again to 16.1% by October 2022 (figure 1A). Although an overall increase in cross-border tobacco purchasing was observed across social grades (table 1), prevalence was higher, and changes over time more pronounced, among social grades ABC1 than C2DE (figure 1B).

**Table 1:** Trends in cross-border and illicit tobacco purchasing prevalence among adults in England who had smoked in the past year

	Prevalence (95% CI)		Prevalence ratioFebruary 2019 to October 2022 (95% CI)
	February 2019*	October 2022*	
Purchased cross-border tobacco in the past 6 months			
All adults who smoked in the past year	5.2% (3.9 to 7.0)	16.1% (12.2 to 21.3)	3.10 (2.03 to 4.73)
ABC1 (more advantaged)	6.6% (4.6 to 9.5)	23.3% (17.0 to 31.9)	3.52 (2.05 to 5.91)
C2DE (less advantaged)	4.4% (2.8 to 7.0)	11.5% (7.1 to 18.6)	2.61 (1.17 to 5.20)
Purchased illicit tobacco in the past 6 months			
All adults who smoked in the past year	9.2% (7.7 to 11.0)	8.5% (6.6 to 11.0)	0.92 (0.70 to 1.21)
ABC1 (more advantaged)	6.8% (5.1 to 9.1)	6.8% (4.9 to 9.6)	1.01 (0.69 to 1.44)
C2DE (less advantaged)	10.6% (8.4 to 13.4)	9.9% (7.0 to 14.0)	0.93 (0.65 to 1.31)

ABC1 includes managerial, professional and upper supervisory occupations. C2DE includes manual routine, semiroutine, lower supervisory and long-term unemployed.

* Weighted prevalence from logistic regression on all adults who smoked in the past year and allowing an interaction between social grade and month (estimates for respondents from social grades ABC1 and C2DE), modelled non-linearly using restricted cubic splines (four knots for cross-border tobacco and three knots for illicit tobacco; see online supplemental material for details of model selection).

The proportion of respondents reporting having purchased illicit tobacco did not change significantly from February 2019 to October 2022 (table 1), with prevalence rising from 9.2% to 14.2% between February 2019 and November 2020, then falling to 8.5% by October 2022 (figure 1C). Prevalence of illicit tobacco purchasing was higher among less advantaged social grades (C2DE compared with ABC1), but time trends did not differ significantly by social grade (table 1; figure 1D).

## Discussion

Between February 2019 and October 2022, there was a non-linear increase in reported cross-border tobacco purchases among adults in England who smoke, with more pronounced changes among those from more advantaged social grades. There was no overall change in the proportion reporting illicit tobacco purchases, nor any difference in trends by social grade.

The curvilinear trend in cross-border tobacco purchasing might be explained by changes in motivation and access resulting from Brexit and the COVID-19 pandemic. Reports of past 6-month cross-border tobacco purchasing tripled between February 2019 and April 2020, with the raw data points indicating a sharp rise in Q2-2020. It is possible this was due to people thinking cross-border tobacco would be cheaper as a result of the UK leaving the EU in January 2020 (despite duty-free purchasing not being implemented until the end of the transition period in January 2021^13^). Alternatively, it could be that people who were travelling in January to March 2020 thought it wise to stock up on tobacco as the impact of COVID-19 on future travel became evident. After the pandemic reached the UK and restrictions on international travel were implemented, past 6-month cross-border tobacco purchases declined substantially, then rebounded rapidly from September 2021 as people began travelling abroad again during the summer of 2021.^27^ The prevalence of cross-border tobacco purchasing was higher, and changes over time were more pronounced, among respondents from more advantaged versus less advantaged social grades. This is consistent with advantaged groups being more likely to more frequently travel overseas than those with lower incomes,^28^ providing greater opportunity to purchase cheaper tobacco abroad.

Illicit tobacco purchasing showed less variability over time, rising by ~50% between February 2019 and November 2020 and returning to baseline levels by October 2022. This suggests that: (1) the COVID-19 pandemic and restrictions on social interaction did not substantially reduce access to illicit tobacco; and (2) as of October 2022, the proportion of adults who smoke buying from illicit sources has not (yet) increased in response to the cost of living crisis. While prevalence of illicit tobacco purchasing was higher among people from less advantaged versus more advantaged social grades, time trends were similar, showing no evidence of increased use of illicit tobacco among those with lower disposable incomes as economic pressures heightened.

A rise in cross-border tobacco purchasing is a cause for concern given people who use cheap tobacco are less likely to try to quit smoking.^6^ Policy measures that reduce access to cheaper sources of tobacco could help increase the rate of quit attempts among those who smoke and accelerate progress towards the government’s Smokefree 2030 target. Reducing duty-free allowances (ideally to zero) and better enforcement of the existing rules around duty-free purchasing are important for driving down the use of duty-free tobacco. Recent reductions in trading standard budgets in England have limited their capacity to tackle illicit tobacco.^29^ These should be reversed and dedicated to local enforcement activity. A low-cost tobacco retailer registration scheme with sanctions could be implemented to provide additional funds for enforcement and detailed surveillance of legal tobacco retailers.^29 30^

This study had several limitations. Data on cross-border and illicit tobacco purchasing were self-reported and related to past 6-month purchases, introducing scope for reporting and recall bias. Participants were not asked about the frequency or quantity of cross-border or illicit tobacco purchasing so we were not able to distinguish between occasional and regular use of these price-minimising strategies. Data collection switched from face-to-face to telephone interviews in April 2020. While this was unavoidable due to the pandemic, it is possible that it contributed to changes we observed; for example, the spike in reports of cross-border tobacco purchasing in spring 2020. However, the fact that we did not see a comparable change in reports of illicit tobacco purchasing does not point to there having been an impact on responses to the source of purchase question. Our models did not account for seasonal variation in cross-border or illicit tobacco purchasing. While visual inspection of the data (figure 1) did not suggest a strong seasonal pattern, estimates of changes in prevalence from the start to end of this period should be considered in light of these data having been collected in different calendar months. Finally, while the sample was nationally representative, participants were recruited from households, meaning people experiencing homelessness—who have much higher smoking prevalence^31^ and who regularly smoke illicit tobacco^32^—are not captured, which may underestimate illicit tobacco purchasing.

In conclusion, despite a fall in cross-border tobacco purchasing during the first year of the COVID-19 pandemic, the proportion of people in England reporting cross-border tobacco purchases is now three times higher than it was at the start of 2019. The proportion reporting illicit tobacco purchases has not changed substantially.

## supplementary material

10.1136/tc-2023-057991 online supplemental file 1
