# Supplementary material for: Trends in cross-border and illicit tobacco purchases among people who smoke in England, 2019–2022
Source: Tob Control. 2023 Jul 18;33(5):e057991. doi: 10.1136/tc-2023-057991 (PMC11503207; doi:10.1136/tc-2023-057991)
Supplement: online supplemental file 1 [file tc-33-5-s001.pdf]

Trends in cross-border and illicit tobacco purchases among people who smoke in England, 2019-2022

Supplementary Material

Part 1: Comparison of models using three versus four knots

Figure S1. Percentage of adults in England who smoked in the past year and who reported purchasing cross-border and illicit tobacco, February 2019 to October 2022: modelled using restricted cubic splines with three knots

Figure S2. Percentage of adults in England who smoked in the past year and who reported purchasing cross-border and illicit tobacco, February 2019 to October 2022: modelled using restricted cubic splines with four knots

Table S1. Comparison of model fit: three versus four knots

Part 2: Best fitting models plotted against monthly data points

Figure S3. Percentage of adults in England who smoked in the past year and who reported purchasing cross-border and illicit tobacco, February 2019 to October 2022: best fitting models with raw weighted monthly data points

Part 3: Analyses of cheap tobacco purchasing (cross-border and illicit combined)

Table S2. Comparison of model fit (cheap tobacco): three versus four knots

Table S3. Trends in cheap tobacco purchasing prevalence among among adults in England who had smoked in the past year

Figure S4. Percentage of adults in England who smoked in the past year and who reported purchasing cheap (cross-border or illicit) tobacco, February 2019 to October 2022

Figure S5. Percentage of adults in England who smoked in the past year and who reported purchasing cheap (cross-border or illicit) tobacco, February 2019 to October 2022: best fitting models with raw weighted monthly data points

Table S4. Trends in cheap tobacco purchasing prevalence among adults in England who had smoked in the past year: log-binomial regression results

Figure S6. Percentage of adults in England who smoked in the past year and who reported purchasing cheap (cross-border or illicit) tobacco, February 2019 to October 2022: log-binomial regression models

Figure S7. Percentage of adults in England who smoked in the past year and who reported purchasing cheap (cross-border or illicit) tobacco, February 2019 to October 2022: log-binomial regression models with raw weighted monthly data points

Part 1: Comparison of models using three versus four knots

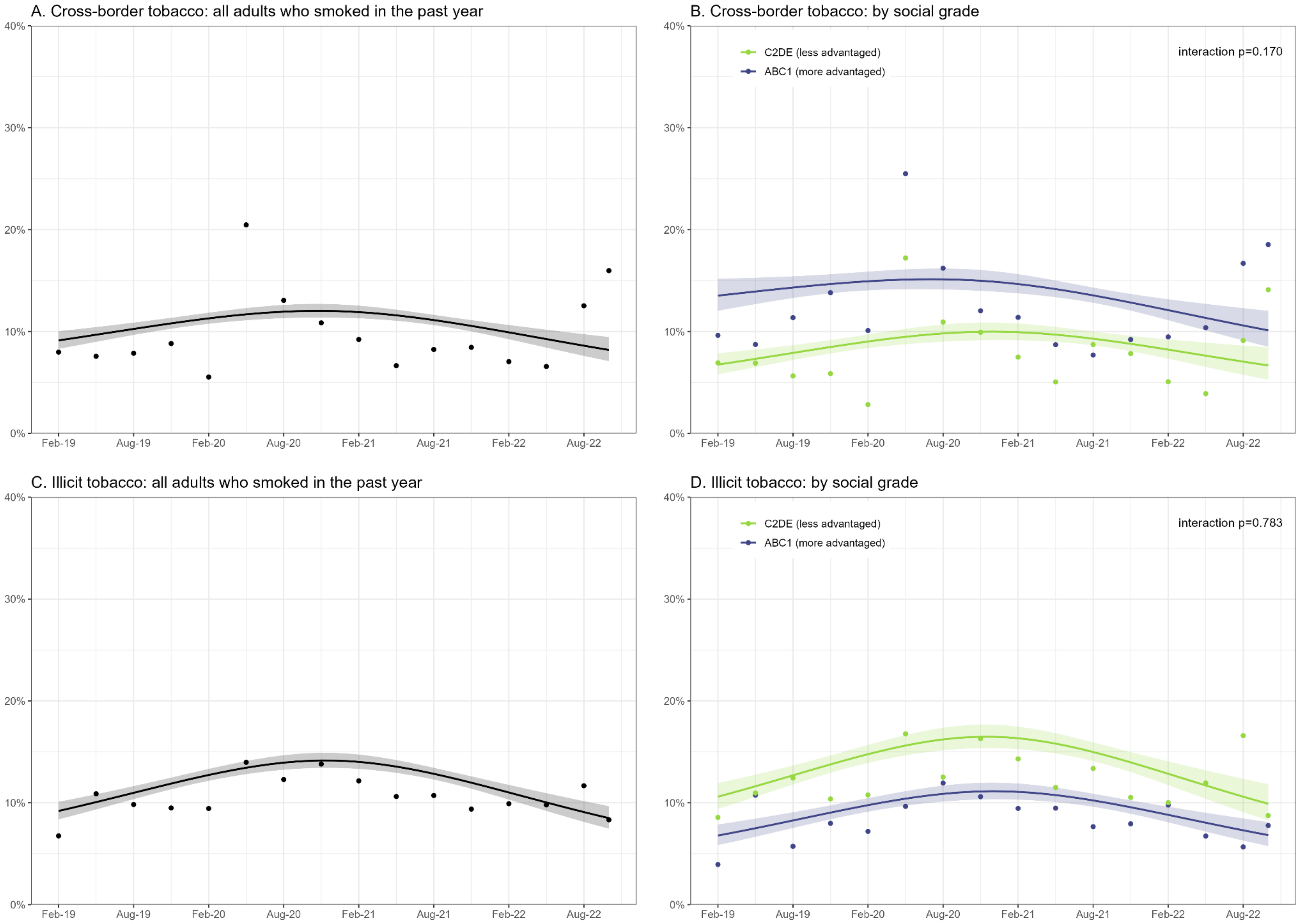

Figure S1. Percentage of adults in England who smoked in the past year and who reported purchasing cross-border and illicit tobacco, February 2019 to October 2022: modelled using restricted cubic splines with three knots. Data are presented for all adults who smoked in the past year (left panel) and by social grade (right panel). Lines represent point estimates from logistic regression with survey month modelled non-linearly using restricted cubic splines (three knots). Shaded areas represent standard errors. Points represent raw weighted prevalence by quarter.

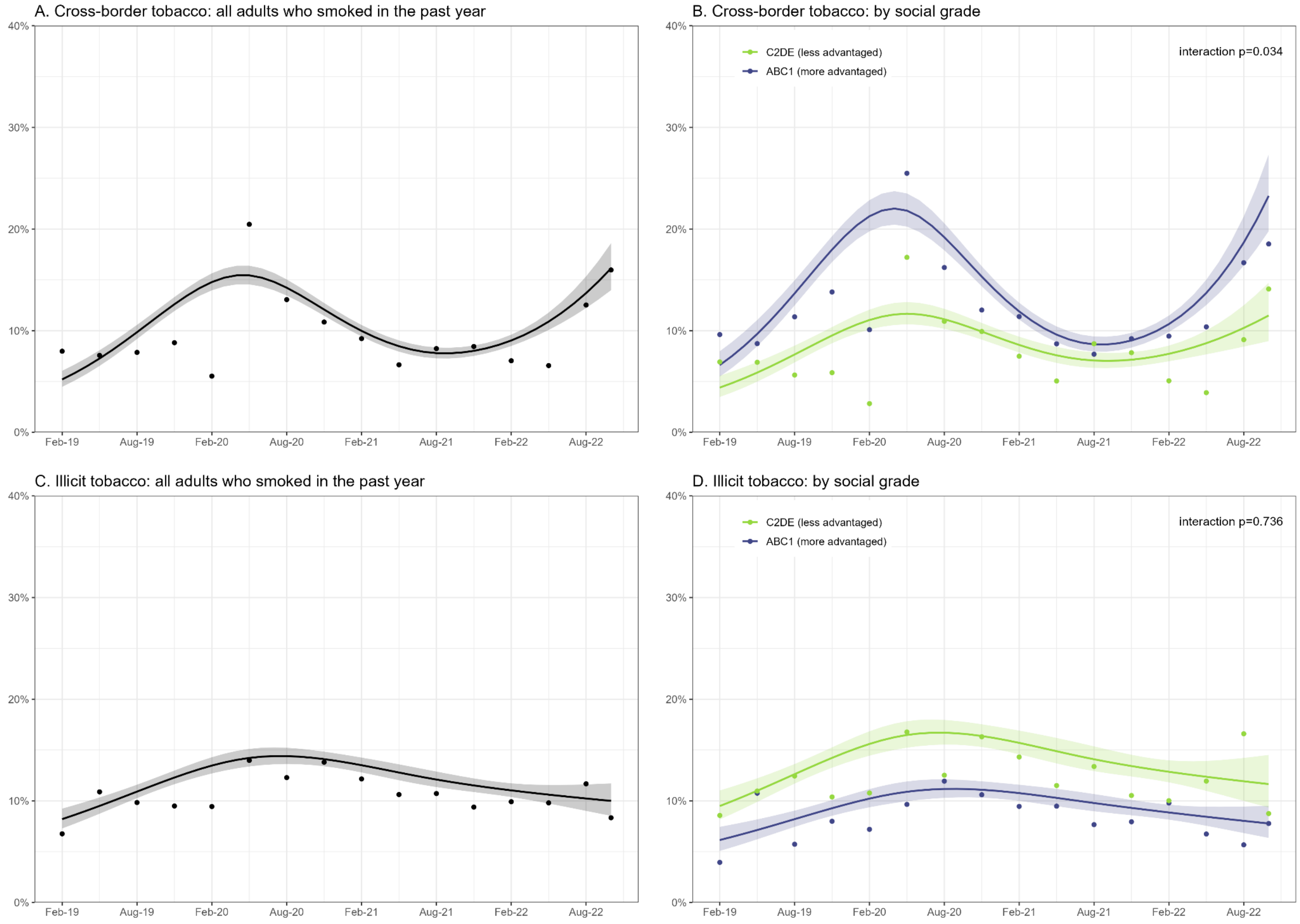

Figure S2. Percentage of adults in England who smoked in the past year and who reported purchasing cross-border and illicit tobacco, February 2019 to October 2022: modelled using restricted cubic splines with four knots. Data are presented for all adults who smoked in the past year (left panel) and by social grade (right panel). Lines represent point estimates from logistic regression with survey month modelled non-linearly using restricted cubic splines (three knots). Shaded areas represent standard errors. Points represent raw weighted prevalence by quarter.

Table S1. Comparison of model fit: three versus four knots

	AIC		
	**3 knots**	**4 knots**	**Difference**
Cross-border tobacco, all adults who smoked in the past year	7167.83	7100.86	-66.97
Cross-border tobacco, by social grade	7122.15	7050.47	-71.68
Illicit tobacco, all adults who smoked in the past year	7686.10	7685.59	-0.51
Illicit tobacco, by social grade	7652.85	7654.91	2.06

AIC, Akaike information criterion. Lower values of AIC indicate better model fit. The criteria for selecting the best fitting model was either the model with the lowest AIC or the simplest model if it was within two units of the model with the lowest AIC score.

Part 2: Best fitting models plotted against monthly data points

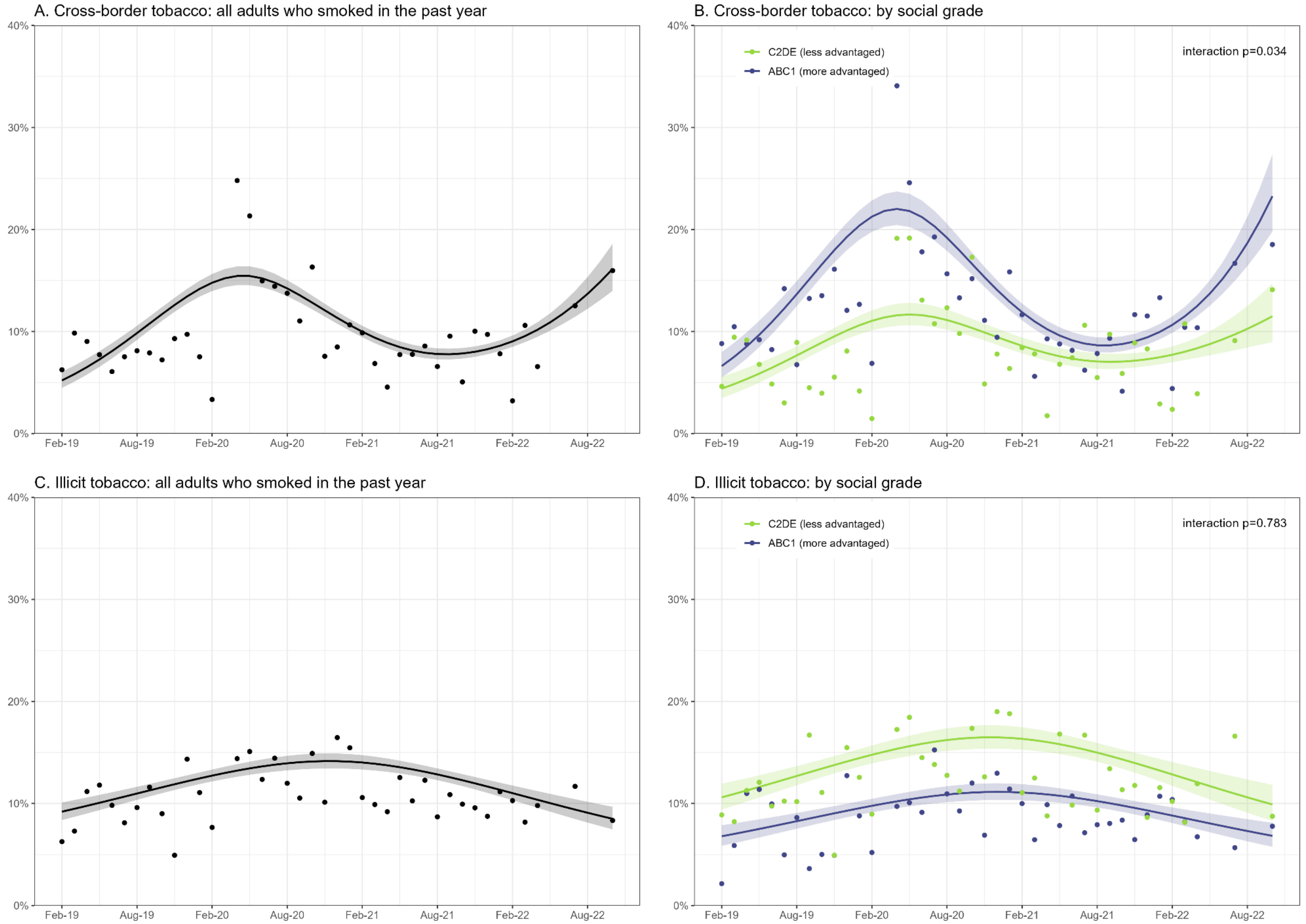

Figure S3. Percentage of adults in England who smoked in the past year and who reported purchasing cross-border and illicit tobacco, February 2019 to October 2022: best fitting models with raw weighted monthly data points. Data are presented for all adults who smoked in the past year (left panel) and by social grade (right panel). Lines represent point estimates from logistic regression with survey month modelled non-linearly using restricted cubic splines (with four knots for cross-border tobacco and three knots for illicit tobacco; see Table S1 for details of model selection). Shaded areas represent standard errors. Points represent raw weighted prevalence by month.

Part 3: Analyses of cheap tobacco purchasing (cross-border and illicit combined)

Table S2. Comparison of model fit (cheap tobacco): three versus four knots

	AIC		
	**3 knots**	**4 knots**	**Difference**
Cheap tobacco, all adults who smoked in the past year	10525.89	10482.39	-43.50
Cheap tobacco, by social grade	10531.50	10484.27	-47.23

AIC, Akaike information criterion. Lower values of AIC indicate better model fit. The criteria for selecting the best fitting model was either the model with the lowest AIC or the simplest model if it was within two units of the model with the lowest AIC score.

Table S3. Trends in cheap tobacco purchasing prevalence among adults in England who smoked in the past year

	**Prevalence [95% CI]**		**Prevalence ratio Feb 19 – Oct 22 [95% CI]**
	**February 2019[1]**	**October 2022[1]**	
**Purchased cheap tobacco in past 6 months**			
All adults who smoked in the past year	13.4% [11.0-16.3]	25.9% [20.6-32.5]	1.93 [1.41-2.65]
ABC1 (more advantaged)	12.4% [9.3-16.5]	32.4% [24.7-42.5]	2.61 [1.67-3.92]
C2DE (less advantaged)	14.2% [10.9-18.4]	21.7% [15.1-31.0]	1.53 [0.91-2.44]

[1] Weighted prevalence from logistic regression on all adults who smoked in the past year and allowing an interaction between social grade and month (estimates for those from social grades ABC1 and C2DE), modelled non-linearly using restricted cubic splines (four knots; see Table S2 for details of model selection).

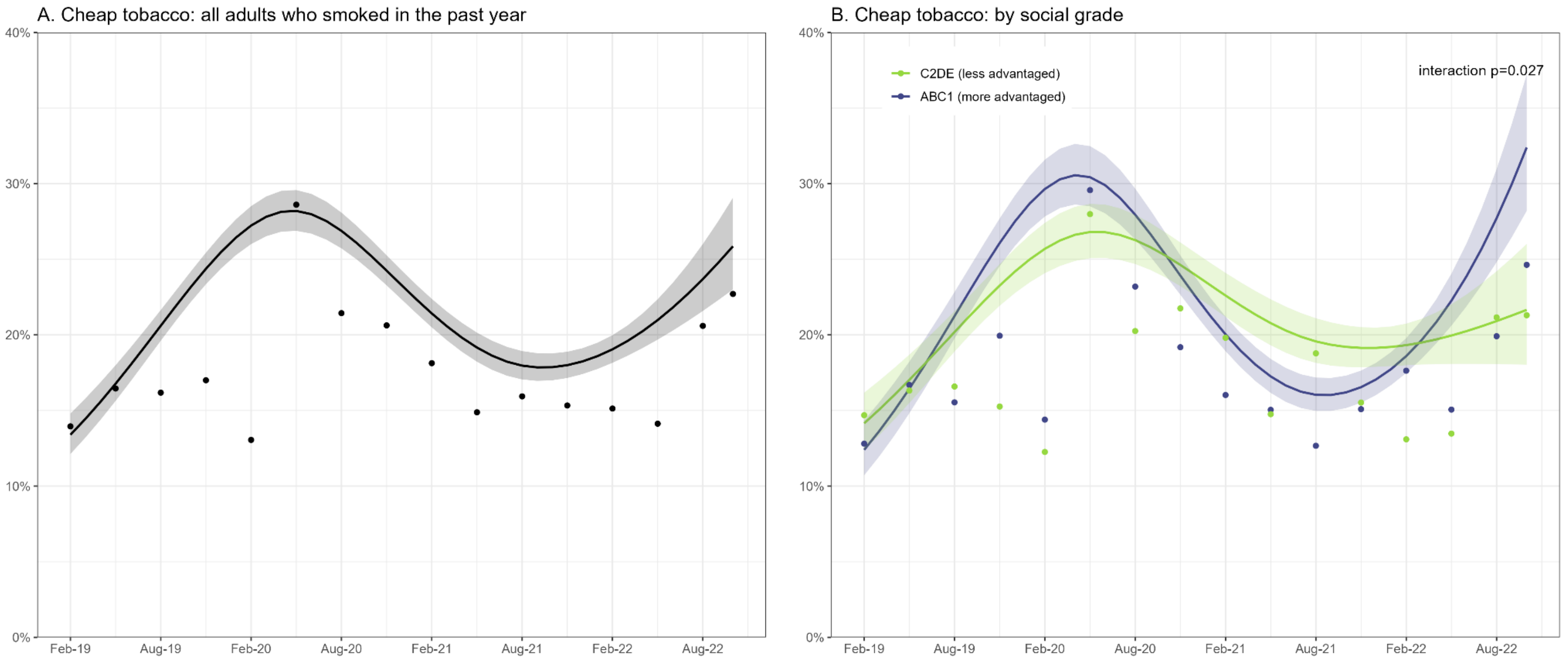

Figure S4. Percentage of adults in England who smoked in the past year and who reported purchasing cheap (cross-border or illicit) tobacco, February 2019 to October 2022. Data are presented for all adults who smoked in the past year (left panel) and by social grade (right panel). Lines represent point estimates from logistic regression with survey month modelled non-linearly using restricted cubic splines (with four knots; see Table S2 for details of model selection). Shaded areas represent standard errors. Points represent raw weighted prevalence by quarter (see Figure S5 for figures showing raw weighted prevalence by month).

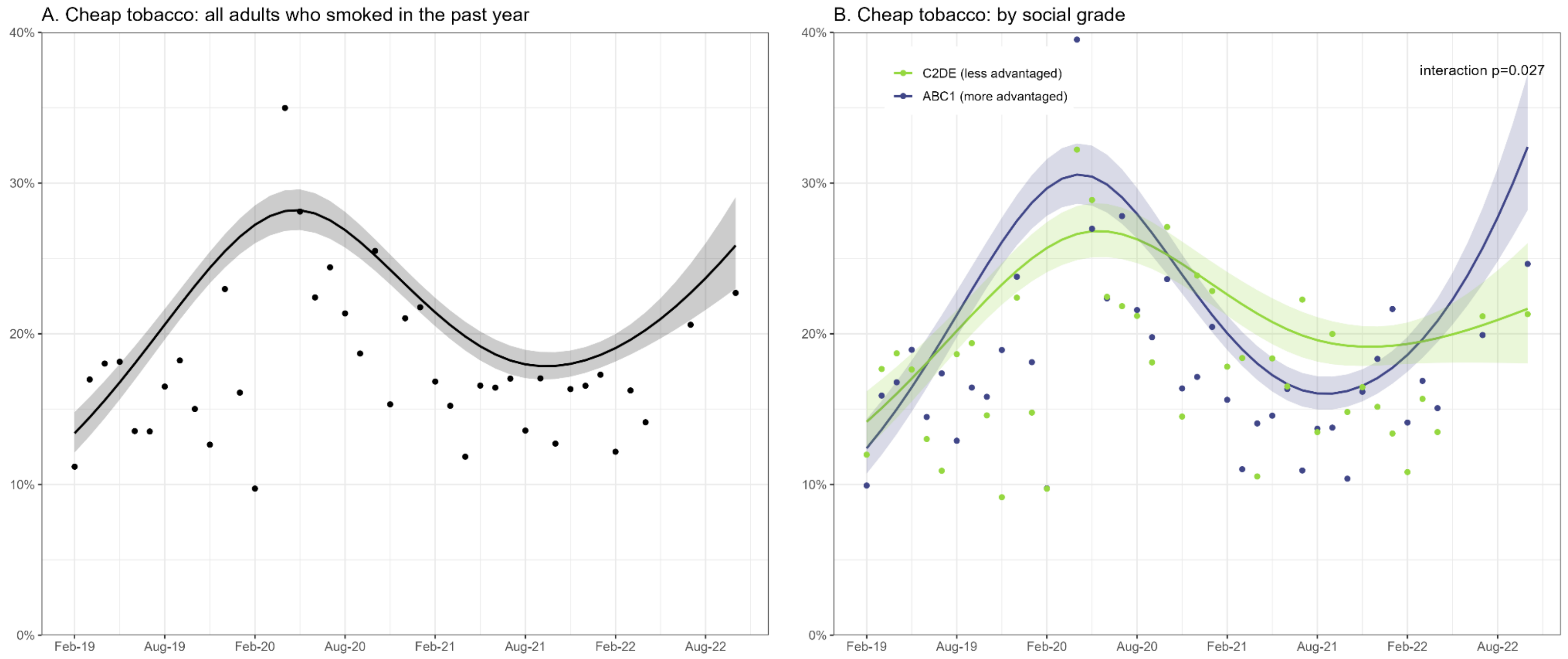

Figure S5. Percentage of adults in England who smoked in the past year and who reported purchasing cheap (cross-border or illicit) tobacco, February 2019 to October 2022: best fitting models with raw weighted monthly data points. Data are presented for all adults who smoked in the past year (left panel) and by social grade (right panel). Lines represent point estimates from logistic regression with survey month modelled non-linearly using restricted cubic splines (with four knots; see Table S2 for details of model selection). Shaded areas represent standard errors. Points represent raw weighted prevalence by month

Note: visual inspection of plotted estimates from logistic regression models against raw data points (Figures S4, S5) indicated the models were overestimating prevalence. We therefore reran these models using log-binomial regression. Results are shown in Table S4 and Figures S6 (against quarterly data points) and S7 (against monthly data points)

Table S4. Trends in cheap tobacco purchasing prevalence among adults in England who smoked in the past year: log-binomial regression results

	Prevalence [95% CI]		Prevalence ratio Feb 19 – Oct 22 [95% CI]
	**February 2019[1]**	**October 2022[1]**	
**Purchased cheap tobacco in past 6 months**			
All adults who smoked in the past year	11.8%	20.6%	1.75 [1.33-2.31]
ABC1 (more advantaged)	11.1%	24.6%	2.23 [1.56-3.13]
C2DE (less advantaged)	12.4%	17.8%	1.44 [0.93-2.12]

[1] Weighted prevalence from log-binomial regression on all adults who smoked in the past year and allowing an interaction between social grade and month (estimates for those from social grades ABC1 and C2DE), modelled non-linearly using restricted cubic splines (four knots).

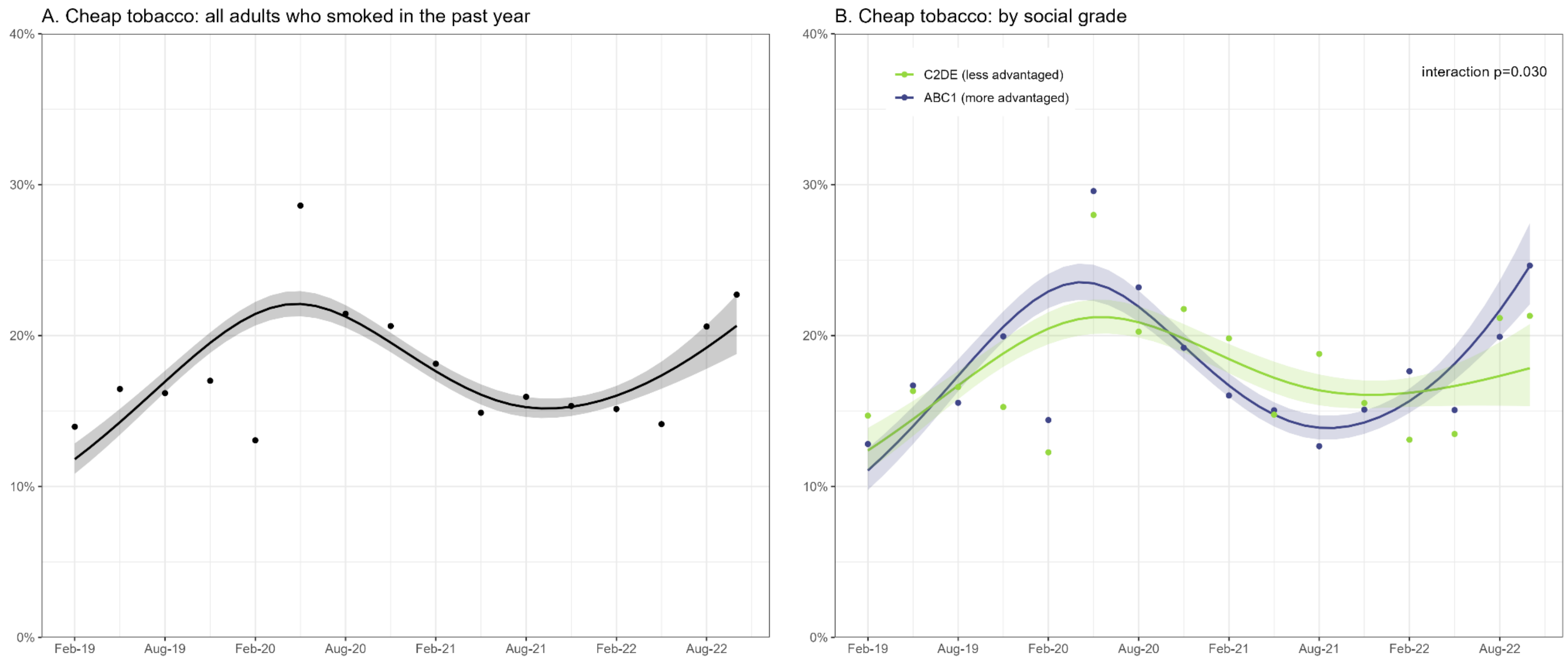

Figure S6. Percentage of adults in England who smoked in the past year and who reported purchasing cheap (cross-border or illicit) tobacco, February 2019 to October 2022: log-binomial regression models. Data are presented for all adults who smoked in the past year (left panel) and by social grade (right panel). Lines represent point estimates from log-binomial regression with survey month modelled non-linearly using restricted cubic splines (with four knots). Shaded areas represent standard errors. Points represent raw weighted prevalence by quarter (see Figure S7 for figures showing raw weighted prevalence by month).

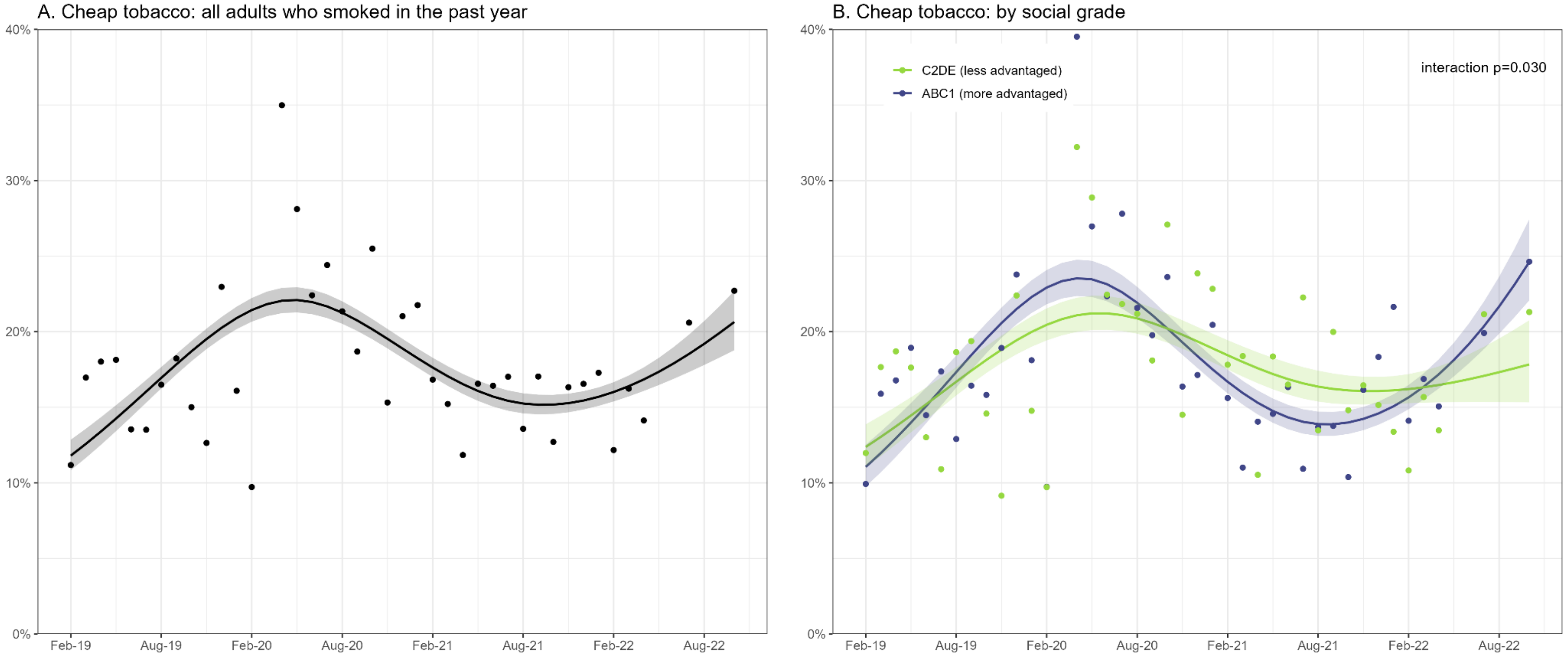

Figure S7. Percentage of adults in England who smoked in the past year and who reported purchasing cheap (cross-border or illicit) tobacco, February 2019 to October 2022: log-binomial regression models with raw weighted monthly data points. Data are presented for all adults who smoked in the past year (left panel) and by social grade (right panel). Lines represent point estimates from log-binomial regression with survey month modelled non-linearly using restricted cubic splines (with four knots). Shaded areas represent standard errors. Points represent raw weighted prevalence by month.
